# Spectral Domain Optical Coherence Tomography in the Diagnosis and Management of Vitreoretinal Interface Pathologies

**DOI:** 10.1155/2012/876472

**Published:** 2012-06-04

**Authors:** Yoreh Barak, Mark A. Ihnen, Shlomit Schaal

**Affiliations:** Department of Ophthalmology and Visual Sciences, University of Louisville School of Medicine, Kentucky Lions Eye Institute, 301 East Muhammad Ali Boulevard, Louisville, KY 40202, USA

## Abstract

The introduction of spectral domain optical coherence tomography (SD-OCT) has enhanced Vitreoretinal Interface (VRI) imaging considerably and facilitated the diagnosis, followup, prognosis determination, and management of VRI-associated pathologies. HR-OCT became a common practical tool seen in almost every ophthalmology practice. Knowledge of SD-OCT image interpretation and recognition of pathologies are required for all ophthalmologists. This paper methodically reviews the normal aging process of the VRI and discusses several commonly encountered VRI pathologies. The role of SD-OCT imaging in VRI-associated disorders such as posterior vitreous detachment, vitreomacular traction syndrome, idiopathic epiretinal membranes, lamellar holes, pseudoholes, and full thickness macular holes is portrayed. Future perspectives of new OCT technologies based on SD-OCT are discussed.

## 1. The Vitreoretinal Interface

**Figure 1:**
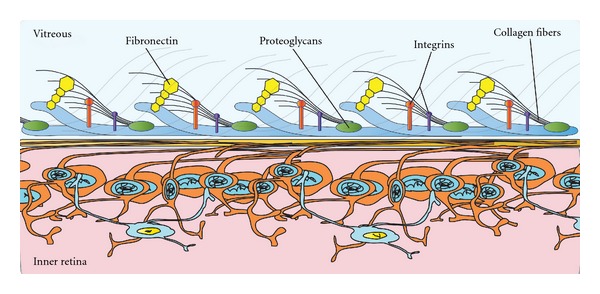
Illustration of the vitreoretinal attachments at the vitreoretinal interface. The posterior vitreous cortex is attached to the ILM by collagen fibers at the vitreoretinal interface. These fibers fuse with the ILM and along with macromolecules, such as laminin, fibronectin, and chondroitin anchor the vitreous cortex to the retina.

The vitreoretinal interface (VRI) is a complex composite structure connecting the vitreous cortex and the inner retina as illustrated in Figure 1.

The posterior vitreous cortex is 100 *μ*m thick. It consists of densely packed collagen fibrils [1] that insert superficially into the internal limiting membrane (ILM) of the retina [2] and attach to the ILM by glue-like macromolecules, such as laminin, fibronectin, chondroitin, and heparan sulphate proteoglycans [3]. The strongest vitreoretinal adhesions have been described at the optic disc, over the retinal blood vessels and at the macular area [3]. This intimate relationship between cortical vitreous and the retina triggers many of the frequently encountered macular pathologies. 

Aging of the human vitreous is characterized by gel liquefaction and the development of fluid-filled pockets, typically beginning in front of the macula and in the central vitreous cavity [4] This progressive process may begin as early as the second decade and eventually leads to degeneration of the vitreous gel and weakened vitreo-retinal adhesion. As the collagen-hyaluronate complexes composing the vitreous progressively degenerate, liquefied vitreous pockets enlarge and enter the posterior hyaloid space. Collapse of the vitreous gel, known as syneresis, leads to a complete PVD with time [2].

Optical coherence tomography (OCT), introduced in the 1990's, is a noninvasive in vivo ophthalmic imaging technique [5]. OCT is based on the principal of Michelson interferometry [6]. Interference patterns produced by low coherence light reflected from ocular tissues are processed into an “A-scan” signal. Multiple A-scan signals are aligned to produce a “B-scan” two-dimensional image that can be thought of as a form of “in vivo histology [6].” In 2002, the time-domain OCT, which was based on a moving reference mirror, became commercially available with an axial resolution of 10 *μ*m.

Since 2004, higher resolution spectral-domain OCT (SD-OCT) has entered clinical ophthalmic practice. SD-OCT is not limited by moving parts. SD-OCT relies on a spectrometer and high-speed camera using the mathematical premise of Fourier transformation for analysis of the reflected light. This results in a significant increase in the amount of data acquired during each session while reducing motion artifacts with an increased signal-to-noise ratio when compared with time-domain OCT. Commercial available SD-OCT machines have a reported axial resolution of 5 to 7 microns [7]. There are some prototypes achieving 3 microns of axial resolution [7].

**Figure 2:**
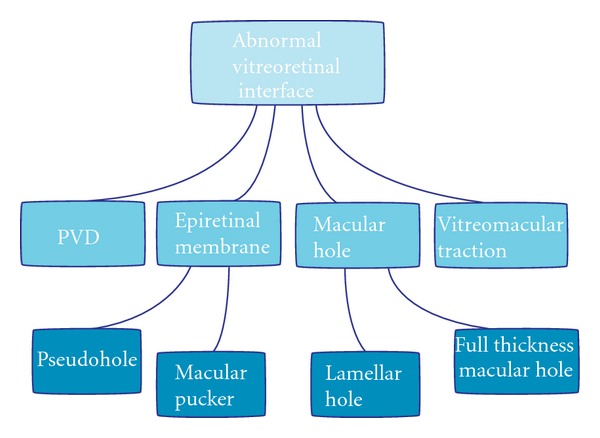
Vitreoretinal-interface-associated pathologies.

This cross-sectional imaging technology has allowed investigators to study and manage patients with VRI disease processes that were previously unrecognizable by biomicroscopy alone. OCT was the primary contributor to our understanding of the pathogenesis and the anatomical sequence of events that underlie VRI pathologies. This revolutionary imaging modality is now prevalent in all ophthalmology clinic settings as it greatly enhances physicians' ability to recognize and diagnose VRI pathologies. This paper reviews the uses of SD-OCT in the evaluation, followup, and management of VRI-associated disorders such as posterior vitreous detachment, vitreomacular traction syndrome, idiopathic epiretinal membranes, lamellar holes, pseudoholes, and full thickness macular holes as illustrated in Figure 2. 

## 2. Posterior Vitreous Detachment

**Figure 3:**
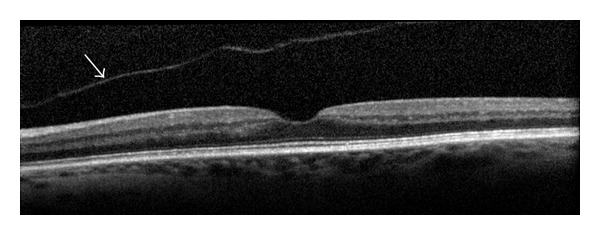
Posterior vitreous detachment (arrow).

Posterior vitreous detachment (PVD) is defined as a separation between the posterior vitreous cortex and the ILM of the retina (Figure 3). 

Aging of the vitreous and syneresis leads to a complete PVD with time [2]. The most commonly associated clinical symptom is the development of floaters. With aging, complete PVD becomes more common with a 10% prevalence in people under the age of 50 and up to 63% of people over the age of 70 [8]. The most common complications of PVD are retinal tears, vitreous hemorrhage, rhegmatogenous retinal detachment, and retinal or optic disc hemorrhage [2, 6, 9]. These complications are mainly caused by dynamic vitreous traction on focal areas of firm vitreoretinal adhesion [9].

For many years PVD could only be diagnosed clinically using biomicroscopy and was believed to be an acute event. Subsequently, ultrasonography was used as the main imaging modality for documentation of PVD [2]. Despite its relatively gross resolution (1 mm) it is nonetheless a reliable tool to determine the presence of a PVD [10]. It is indeed advantageous in eyes with media opacities such as corneal opacities, dense cataracts, vitreous hemorrhage, or vitreous inflammation.

**Figure 4:**
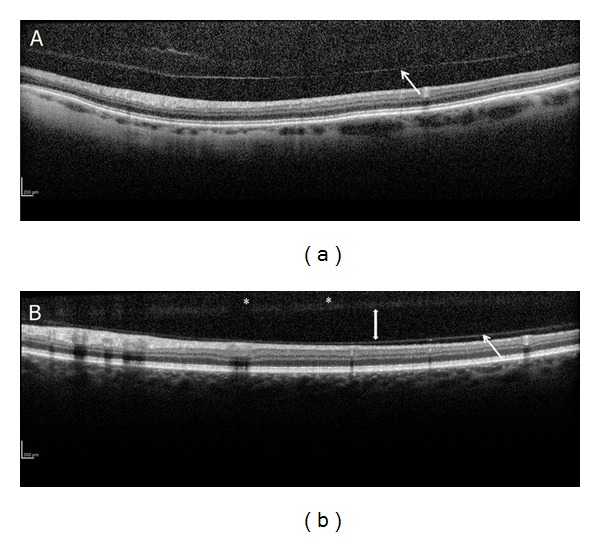
SD-OCT images of PVD and vitreoschisis. PVD; posterior hyaloid (arrow) separated from the retina (a). Vitreoschisis with liquefaction (double-head arrow) of the vitreous between a formed vitreous (asterisk) and an attached posterior hyaloid (arrow).

SD-OCT enables us, in our day-to-day practice, to image and to study the posterior hyaloid face and its intimate relationship with the retina. It allows not only early diagnosis of vitreous pathologies but also the ability to differentiate PVD from other clinical entities, such as vitreoschisis (Figure 4). The SD-OCT is a reliable and an objective tool to assist the clinician in making the correct diagnosis and determining treatment options. For example, an observation of vitreomacular traction syndrome following an acute traumatic incomplete PVD that was recently demonstrated by SD-OCT may be transient in nature, allowing the option of observation and followup by SD-OCT as an alternative to early surgical intervention [11]. A recent study by Johnson [4] used SD-OCT imaging to evaluate the early stages of PVD. It was revealed that PVD is most probably an insidious, chronic event that begins in the perifoveal macula and progressively detaches over a prolonged period of time, leaving the foveal and optic nerve attachment to separate last.

The majority of PVDs are asymptomatic and do not require any treatment [4]. Most symptomatic patients experience floaters and predominantly do well with observation alone. Although still controversial, there have been a growing number of recent reports advocating small incision sutureless vitrectomy surgery for the removal of symptomatic floaters [12, 13].

Vitreoschisis and incomplete PVD, determined in part by the size and strength of the residual vitreoretinal adhesion [4], may be complicated by a variety of vitreoretinal interface pathologies in the macular area as discussed below. 

## 3. Vitreomacular Traction Syndrome

The classic form of Vitreomacular traction (VMT) syndrome is characterized by partial PVD with residual strong and focal posterior vitreomacular adhesions. This results in anteroposterior and tangential tractional forces applied by the vitreous to the foveal and parafoveal regions. Based on SD-OCT data, it appears that there are 2 subclasses of vitreomacular traction; focal foveolar adhesion and broad macular adhesion [4, 14, 15]. Decreased visual acuity may result from secondary intraretinal edema and a distortion of the normal macular architecture. Other common symptoms associated with VMT syndrome include metamorphopsia, micropsia, and photopsia.

**Figure 5:**
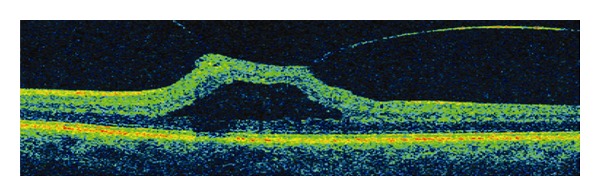
Vitreomacular traction.

Now commercially available, OCT has been tremendously helpful in confirming many cases of VMT that were clinically undetectable (Figure 5). Although conventional time domain OCT was able to demonstrate the vitreoretinal interface, the ability to image the posterior hyaloid membrane was limited by the slow scan speed, limited sensitivity, and poor axial resolution. The increased axial resolution, the augmented signal/noise ratio, and the higher scan rate of new SD-OCT have dramatically improved the ability to visualize the vitreomacular interface and posterior hyaloid membrane [16].

Three-dimensional scans made possible with SD-OCT have advanced the comprehensive evaluation of the vitreoretinal interface providing additional clinically significant information. The 3-dimensional reconstruction modality enables meticulous presurgical planning, with the potential for improved postsurgical outcomes [16]. Koizum et al. [14] performed a three-dimensional evaluation of the VRI in VMT syndrome using SD-OCT. They found that most of the eyes with VMT syndrome had concurrent epiretinal membranes. These epiretinal membranes increase the adhesion between the vitreous and the retina and serve as an anchor for the cortical vitreous on the inner surface of the retina therefore preventing spontaneous separation of the vitreous from the macula.

SD-OCT has triggered focused attention to special conditions in which VRI pathologies develop. For instance, a complete PVD is less prevalent in diabetic patients with clinically significant diabetic macular edema compared to diabetic patients without diabetic macular edema [17]. It is well established that diabetic macular edema may be exacerbated by the vitreomacular traction effects of partial vitreous detachment [18]. A recent study using SD-OCT to assess the VRI in eyes with diabetic macular edema, epiretinal membranes, and incomplete PVD [19] found that the posterior cortical vitreous and the hyper-reflective adherent membrane, which is generally designated as an epiretinal membrane, appeared as one continuous thick membrane. Based on these novel observations which were supported by histologic findings [20], it can be assumed that the hyperreflective adherent epimacular membrane is commonly composed of an integrated fibrocellular membrane from the epiretinal membraneposterior hyaloid complex. 

In daily practice, in addition to confirming the diagnosis of VMT, SD-OCT plays an important role in following patients with VMT and in determining their visual prognosis. In some cases, spontaneous resolution of traction may occur, justifying a period of clinical observation before surgical intervention [21, 22]. For symptomatic patients, small gauge vitrectomy surgery and release of the vitreous traction may be easily and safely applied [23, 24]. It is important to keep in mind that clinical and histologic studies have shown that residual cortical vitreous commonly adheres to the inner retinal surface following vitrectomy despite peeling of the posterior hyaloids [25]. Cortical postoperative vitreous remnants may organize into a fibrocellular epiretinal membrane with subsequent contraction causing macular pucker [26]. Other limitations of vitrectomy include surgical complications such as retinal tears, retinal detachments, cataract formation/progression, and intraocular infection as well as high costs.

Pharmacologic vitreolysis, applied as an intravitreal injection, is an emerging possible treatment for persistent vitreomacular-adhesion-related pathologies [27].

Over the past 15 years, investigators have increasingly examined alternative methods for PVD induction which focused on the use of pharmacologic agents to modify the molecular structure of the vitreous thereby eliminating its role in the pathogenesis of retinal diseases. While early interest in pharmacologic vitreolysis has focused on its application as an adjunct to vitrectomy surgery and removal of fibrovascular proliferative membranes [28], investigators have quickly realized its potential as a stand-alone therapy [29]. Vitreolytic agents may potentially improve anatomical and functional outcomes in VMT patients. They may also be used as a prophylactic measure in conditions in which PVD is associated with an improved prognosis (DME) [27]. 

Results from an initial clinical trial evaluating the safety and preliminary efficacy of vitreolysis with intravitreal microplasmin in patients suffering from VMT show the drug to be well tolerated and capable of inducing a pharmacologic PVD in some patients [30].

The ancillary benefits of pharmacologic vitreolysis include: decreased costs, based on shorter surgical times or decreased incidence of progressive disease requiring surgery; greater access to therapy, based on the simple instrumentation involved in the injection administration and a possible future transition to office-based procedures [31]. Furthermore, these agents will reduce patient exposure to inherent vitrectomy-related complications, such as endophthalmitis, cataract formation, iatrogenic retinal breaks and anesthesia related complications [27]. 

## 4. Full Thickness Macular Hole

**Figure 6:**
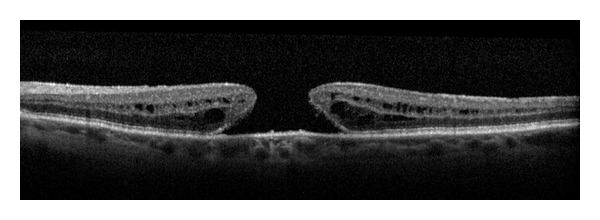
Full thickness macular hole.

Full thickness macular hole (FTMH) is a vertical split in the foveal neurosensory retina (Figure 6). It is more common in females and occurs primarily in the sixth to eighth decades of life. The risk of developing a macular hole in the other eye of patients with unilateral macular hole has been reported to be 11–13% overall [32]; however, it increases to 50% if a premacular hole configuration is noted on OCT [33].

The etiology of macular hole formation is still unknown. SD-OCT data gathered in recent years support the hypothesis that vitreoretinal abnormalities and vitreomacular traction play a major role in idiopathic macular hole formation [32, 34]. Visual symptoms include metamorphopsia and diminished central visual acuity ranging from 20/40 to 5/200 in later stages.

Clinically, a FTMH might be confused with other VRI disorders such as a pseudohole and lamellar hole (discussed below). SD-OCT helps the clinician distinguish between these VRI conditions and has improved our understanding of the role of vitreoretinal adhesions in the pathogenesis of these disorders. The conventional management of FTMH is surgical treatment. Initially advocated in 1991 by Kelly and Wendel [35], vitrectomy with gas tamponade and face down positioning is currently the treatment of choice for FTMH. Variations of this procedure have evolved over the years to include ILM peeling, with or without adjuvant staining [36]. Recent data questions the need for face down positioning after surgery for small (<400 *μ*m) FTMH [37, 38]. More recently, pharmacological vitreolysis has been recommended for the closure of small FTMH [30].

**Figure 7:**
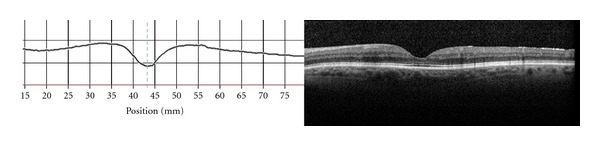
Premacular hole configuration SD-OCT (right) and foveal thickness graph (left) illustrating steep and asymmetrical slopes.

In day to day retina clinic, SD-OCT is an invaluable tool in the pre- and postoperative diagnosis and followup of patients with FTMH. Newly, a mathematical analog of the premacular hole foveal anatomic configuration was first described to enable recognition of patients prone to developing FTMH (as seen in Figure 7) [33]. Using OCT foveal thickness maps, the mathematical analog of a premacular hole, foveal anatomic configuration was found to be significantly different from the normal foveal configuration and was composed of a steep nonsymmetrical foveal slope with a wide fovea on OCT scans. Fifty percent of the patients with a premacular hole configuration consequently developed bilateral macular holes. This high incidence compared to a previously reported incidence of 11% to 13% of bilateral macular holes [32] may indicate that this foveal configuration predisposes a subset of high risk patients to develop bilateral macular holes [33]. The exciting new clinical ability to identify this distinct macular configuration may allow early diagnosis, close followup and better management of macular hole-prone patients as seen in Figure 7 [33].

SD-OCT is also used to determine the prognosis for vision recovery by assessing the structural integrity of the photoreceptors before and after macular hole surgery by imaging of the inner segment/outer segment photoreceptor (IS/OS junction) defect in patients with macular holes. The extent of the IS/OS junction defect can be a prognostic feature for the visual outcome after macular hole surgery [39, 40]. A study evaluating the ability of SD-OCT images of the IS/OS junction to predict macular hole surgery outcomes demonstrated that the mean total area and maximum length of the IS/OS junction defect at 12 months after surgery was significantly and negatively correlated with the postoperative visual acuity. The conclusion of the study was that SD-OCT is useful for quantitatively measuring IS/OS junction defects, and that postoperative IS/OS junction may play an important role in visual recovery after macular hole surgery [40]. 

## 5. Lamellar Hole

**Figure 8:**
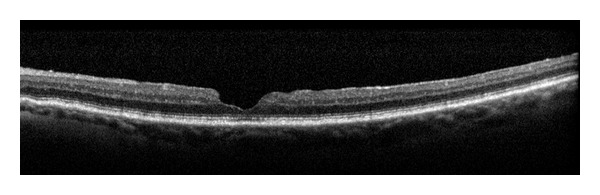
Lamellar hole with intact photoreceptors.

Lamellar hole is a partial thickness defect in the neurosensory retina with intact photoreceptors, as shown in (Figure 8). 

## 6. Pseudohole

**Figure 9:**
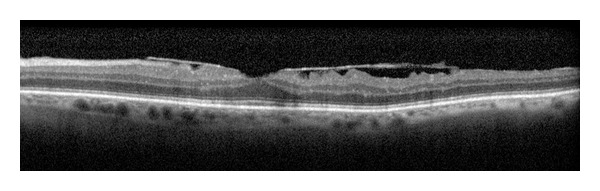
Pseudohole without any neurosensory defects.

Pseudohole is a hole in an epiretinal membrane (ERM) without any neurosensory defects in the retina (Figure 9).

## 7. Epiretinal Membrane

**Figure 10:**
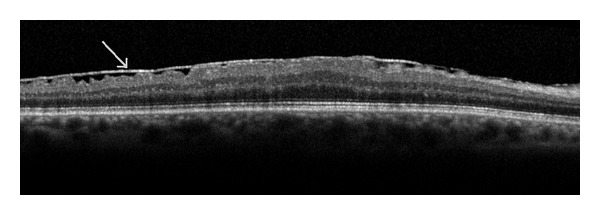
Epiretinal membrane (arrow).

Epiretinal membrane (ERM) or macular pucker is an avascular, fibrocellular membrane on the inner surface of the retina (Figure 10).

ERM results from proliferative changes at the VRI and can be either secondary to other ocular conditions or idiopathic in nature. Secondary membranes are associated with a variety of retinal disorders, including retinal tears, retinal vascular diseases (branch retinal vein occlusion and central retinal vein occlusion), uveitis, trauma, or retinal detachment surgery [1]. Partial or complete PVD has been found in 80% to 95% of eyes with idiopathic ERM [41]. This was suggested to be secondary to vitreous schisis and vitreous remnants on the retina promoting subsequent epiretinal fibrocellular proliferation [42].

Idiopathic premacular membranes have a wide range of severity. They may be quite subtle, causing minimal loss of vision or may result in macular edema and distorted vision caused by traction exerted by the membrane and resultant leakage from the perifoveal capillaries.

In clinical practice SD-OCT has proven useful in the evaluation and treatment of ERMs. Using SD-OCT, one can easily differentiate the posterior hyaloid, a minimally reflective signal, from an ERM, which is highly reflective [43]. OCT has also been helpful in confirming the relationship between PVD and ERM. OCT is valuable for following the natural history of epiretinal membranes [43]. Visually significant ERMs are usually removed surgically resulting in a 2-line improvement on average [44]. Although surgical intervention was previously advised only after significant reduction in visual acuity (<20/60), SD-OCT made it possible to obtain a very accurate image of early changes in the retina, such as ERM-related macular edema and/or distortion of the inner and outer retina allowing early, minimally invasive, small gauge surgical intervention, and possibly better visual outcome.

## 8. Future Perspective

New advancements in the use of high-resolution OCT for retinal imaging are continuously emerging. These practical and experimental tools most probably will have a marked impact on the way we treat our patients in retina practice in the future. One of the most intriguing developments is the experimental use of intraoperative microscope-mounted SD-OCT. The possibility to use SD-OCT intraoperatively provides thrilling new insights into the subtle changes of retinal anatomy during the performance of macular surgery. Intra-operative SD-OCT may be a future practical tool for facilitating vitreoretinal surgery [45]. SD-OCT high axial resolution also allows for a three dimensional en face or “C-scan” to be produced offering a new view of the different layers of the retina and the vitreoretinal relationship [7, 46]. Another new development is the Swept source OCT that can achieve ultrahigh axial resolution of 2-3 *μ*m by sweeping a narrow bandwidth light source through a broad optical range [47]. Total retinal blood flow measurement with this newer ultrahigh speed swept source/Fourier domain OCT [47] may enable us to demonstrate and measure the increased blood flow reported after peeling of the posterior hyaloid [48] and may abele us to measure blood flow in occlusive retinal vascular diseases and/or ischemic diseases of the retina.

## 9. Conclusion

SD-OCT has broadened a new horizon in the basic understanding and interpretation of vitreoretinal interface disorders. It allows early diagnosis, better followup, and more intelligent information-based surgical decision making. SD-OCT has recently been used in the screening of patients at risk for vitreoretinal interface disorders, as well as determining and predicting their visual prognosis. With further advancement of this technology, higher resolution OCT carries the potential of perfecting visual outcomes for patients with VRI disorders.
